# Association between Exposure to Endocrine Disruptors in Drinking Water and Preterm Birth, Taking Neighborhood Deprivation into Account: A Historic Cohort Study

**DOI:** 10.3390/ijerph13080796

**Published:** 2016-08-09

**Authors:** Marion Albouy-Llaty, Frédérike Limousi, Camille Carles, Antoine Dupuis, Sylvie Rabouan, Virginie Migeot

**Affiliations:** 1 Department of Analytical Chemistry, Pharmaceutics and Epidemiology, School of Medicine and Pharmacy, University of Poitiers, 6 rue de la Milétrie, 86034 Poitiers Cedex, France; frederike.limousi@gmail.com (F.L.); antoine.dupuis@univ-poitiers.fr (A.D.); sylvie.rabouan@univ-poitiers.fr (S.R.); virginie.migeot@univ-poitiers.fr (V.M.); 2 Health–Endocrine Disruptors-EXposome, Clinical Investigation Center, Inserm U1402, 86021 Poitiers Cedex, France; 3 Teaching Hospital of Poitiers, Biology-Pharmacy-Public Health Pole, 2 rue de la Milétrie, BP577, 86021 Poitiers Cedex, France; 4 Epidemiology of Cancer and Environmental Exposures, Inserm U1219, University of Bordeaux, 146 rue Léo Saignat, 33076 Bordeaux, France; Camille.Carles@isped.u-bordeaux2.fr

**Keywords:** endocrine disruptor, mixture, drinking-water, preterm birth, social inequalities, European deprivation index

## Abstract

*Background*: The relationship between preterm birth (PTB) and endocrine disruptor exposure in drinking-water has only occasionally been studied. The objective of this work was to investigate the relation between exposure to atrazine metabolites, or atrazine/nitrate mixtures, in drinking-water during pregnancy and prevalence of PTB neonates, while taking neighborhood deprivation into account. *Method*: A historic cohort study in Deux-Sèvres, France, between 2005 and 2010 with a multiple imputation model for data of exposure to atrazine metabolites and a logistic regression were carried out. *Results*: We included 13,654 mother/neonate pairs living in 279 different census districts. The prevalence of PTB was 4%. Average atrazine metabolite concentration was 0.019 ± 0.009 (0.014–0.080) µg/L and 39% of mothers lived in less deprived areas. The individual data were associated with risk of PTB. The risk of PTB when exposed to highest concentration of atrazine metabolite adjusted for confounders, was ORa 1.625 95% CI [0.975; 2.710]. Taking, or not, neighborhood deprivation into account did not change the result. Exposure to atrazine/nitrate mixtures remained non-significant. *Conclusions*: Even if we took neighborhood deprivation into account, we could not show a significant relationship between exposure to atrazine metabolites, or mixtures, in drinking-water during the second trimester of pregnancy and PTB.

## 1. Introduction

In France, there are 60,000 preterm births per year and the rate has increased from 5.9% in 1995 to 7.4% in 2010, particularly for births between 32 and 36 weeks of gestation [1]. The consequences of preterm birth on health are important in both the short and the long term [1,2]. Many maternal and fetal characteristics have been associated with preterm birth, including socio-economic, social or psychosocial factors, adverse behaviors, nutritional status, medical factors (obstetric history, infection, uterine contractions and cervical length), biological, genetic markers [3] and environmental factors (air or water pollution) [4,5]. The relationship between preterm birth and material or social deprivation has been widely studied, a mother living in a more deprived neighborhood having more risk of preterm birth [6,7,8,9,10,11,12,13].

Among environmental exposures, exposure to Endocrine Disruptor Compounds (EDCs), such as the herbicide atrazine, is particularly relevant [14], notably in relation with preterm birth. However, the level of evidence is deemed “inadequate” [15,16,17,18,19]. The studies have particularly failed to account for neighborhood deprivation, which may modify the relation between environmental exposure and perinatal outcomes [20,21,22].

We hypothesized that there is a relationship between exposure to atrazine metabolites in drinking water during pregnancy and preterm birth taking the social context (neighborhood deprivation) into account. Our main objective was to explore the relationship between exposure to atrazine metabolites in drinking-water measured at community water systems during pregnancy and the prevalence of preterm birth between 2005 and 2010 in the district of Deux-Sèvres (France) taking neighborhood deprivation into account. The second objective was to explore the same relationship with regard to exposure to nitrate/atrazine metabolite mixtures, as nitrate is also a prevalent EDC [23,24].

## 2. Material and Methods

### 2.1. Study Area

A historic cohort study was carried out in Deux-Sèvres between 2005 and 2010. Deux-Sèvres is a district of the Poitou-Charentes region in western France with an area of 5999 sq.km and a population of 362,944 inhabitants in 2007 with about 4100 births per year. Agricultural activity is paramount and essentially involves livestock, predominantly sheep and goats, along with cereal production. A quarter to half of the drinking water in this district comes from surface waters. In Deux-Sèvres, 12.0% of the population is below the poverty threshold (12.6% in France): 14% in rural areas and 10% in urban areas. It appears to be a heterogeneous socioeconomic district [25].

### 2.2. Individual Data

Individual data (particularly preterm birth) came from birth records of the district office of maternal and childhood protection, via the mandatory health certificates at birth completed by the maternity prior to an infant’s discharge. Validation of the individual data drawn from birth records was carried out according to a methodology approved by the Research, Study, Evaluation and Statistics Directorate—DREES (French Ministry of Social Affairs and Health) [26].

The available individual data were socio-economic factors (parental occupations), social factors (single-parent family), adverse behaviors (smoking during pregnancy), medical factors (number of previous pregnancies, quality of follow-up, caesarian section, medical history of preterm birth in siblings), fetal factors (sex, birth weight) and gestational age (number of weeks of amenorrhea, reported by obstetrical staff at birth), age of mother and place of residence at birth. Mother’s age was divided into three classes: <20 years; 20–35 years; >35 years of age. Number of previous pregnancies was divided into two classes: primiparity and multiparity. Mother’s occupation was classified as disadvantageous (workers and unemployed), moderately advantageous (self-employed, employees and farmers) or advantageous (managers and executives) [27]. Quality of follow up was high if it was in conformity with French health authority guidelines that is to say if at least three ultrasound examinations were carried out, and, before 2006, if there were seven antenatal care examinations during pregnancy or, since 2006, if the first antenatal care examination took place during first trimester. Preterm birth status was defined as birth before 37 weeks of amenorrhea.

### 2.3. Area-Level Data

Area-level socioeconomic status was characterized by the European Deprivation Index (EDI). The geographical units used were IRIS (regrouped statistical information blocks) as defined by the French National Institute for Statistics and Economic Studies (INSEE), an IRIS representing the smallest geographical census unit available in France. The regional capital and other major towns are divided into several IRIS units, and small towns form a single IRIS. Each IRIS includes approximately 2000 individuals with relatively homogeneous social characteristics. In 2007, Deux-Sèvres counted 305 municipalities and 362 IRIS.

The EDI reflects objective and subjective, material and social deprivation [28]. Score of the EDI in France for each IRIS is a sum of weighted variables (percentage of overcrowding houses, exclusive use of bath or shower, foreign nationality, households without a car, no business leaders-company managers/intermediate occupations, lone parent household, low level of education, unemployed, not be owner) for which weights came from multiple logistic regression [28]. EDI was divided into tertiles. The higher the index, the more the IRIS is considered to be deprived [28]. The data needed for its construction were drawn from the population census carried out by INSEE in 2007. Mothers’ addresses were geocoded at the IRIS level through a correlation map effective in 1999. The map was provided by the Maurice Halbwachs Center (Paris, France), which collects surveys and databases following agreements with the INSEE those mothers lived in the district at the time, several ministerial statistical services and other public institutions [29].

### 2.4. Exposure

In the study area, atrazine is the most abundantly used substance sold for corn application, with 125 tons per year, and nitrate sources are mainly from livestock, whereas natural production and human pollution does exist too.

Drinking-water atrazine and atrazine metabolites (desethylatrazine, 2-hydroxyatrazine) came from samples routinely taken at community water systems (CWS) between 1 April 2004 and 31 December 2010 by a laboratory accredited by the Regional Health Agency. This laboratory uses a HPLC-MS-MS method with a limit of quantification (LQ, lowest amount of analyte in a sample which can be detected and quantified) and limit of detection (LD, lowest amount of analyte in a sample which can be detected, but not necessarily quantified).

Exposure of each pregnant woman was defined by assessing samples drawn from the CWS supplying her municipality of residence during trimesters of pregnancy. During a given trimester of pregnancy, each mother-neonate pair had several samples taken (from 1 to 34): 75% of pairs had one sample, 23% two and 2% three. We calculated the mean atrazine metabolite concentration for each trimester. When the mean was above the LQ, atrazine metabolite exposure was defined as positive for the trimester [30]. As atrazine metabolite concentrations are left censored data with repeated measures, the maximum likelihood estimation method has been employed to impute these data [31], according to the LQ (0.05 µg/L between 2004–2006 and 0.02 µg/L between 2007–2010). Exposure of atrazine metabolite was treated as continuous variable and was categorized in three groups.

Mixture exposure was defined by a combination of the two variables: positive exposure to atrazine metabolite when atrazine metabolite concentration by trimester was above the LQ (negative exposure when it was under LQ) and tertiles of nitrates concentration [30]. Nitrate concentrations terciles were constructed in two steps: for each trimester, since the pregnant women had had several samples extracted in their CWS, we calculated the mean nitrate concentration for all samples taken during the trimester. Following that, we described the distribution and categorized it in terciles [30].

It has resulted in six classes: unexposed to atrazine metabolites but exposed to the first tercile of mean nitrate concentrations (P0N1), unexposed to atrazine metabolites but exposed to the second tercile of mean nitrate concentrations (P0N2), unexposed to atrazine metabolites but exposed to the third tercile of mean nitrate concentrations (P0N3), exposed to atrazine metabolites and to the first tercile of mean nitrate concentrations (P1N1), exposed to atrazine metabolites and to the second tercile of mean nitrate concentrations (P1N2), exposed to atrazine metabolites and to the third tercile of mean nitrate concentrations (P1N3) [30].

If multiple mechanisms of preterm birth (immune system response, inflammatory processes, and endocrine mechanisms) [32] can be activated, there may not be a single critical exposure period [33], so we examined exposure to atrazine metabolites and mixture during each trimester except for last trimester because preterm birth could occur before an exposure in the third trimester.

### 2.5. Analysis Dataset

All live births in Deux-Sèvres from 1 January 2005 through 31 December 2010 of neonates whose mothers lived in the district at the time of birth and whose birth certificates had been recorded (*n* = 24,316), that is to say 98% of the declared births, were included in the analyses.

### 2.6. Exclusions to the Dataset Included

Multiple births, early deaths (before birth record completion) and newborns with congenital malformations were excluded. Mothers residing in municipalities supplied by several CWSs, mothers who were exposed to other pesticides in drinking-water, or who had undergone no measurement of nitrate and/or atrazine metabolites during the second trimester of pregnancy, were excluded. Mother/neonate pairs that could not be located in an IRIS unit because they lived in a municipality of which the configuration had changed since 1999 or on account of an incomplete address were also excluded.

### 2.7. Statistical Analysis

We firstly have imputed left censored data of exposure to atrazine metabolite (57% of data) with a maximum likelihood estimation method for repeated measures (PROC NLMIXED) [31].

We examined association between each individual risk factor, neighborhood deprivation, exposure and preterm birth with a logistic regression analysis. We compared exposure to atrazine metabolite according to neighborhood deprivation, rural area and season through chi2 tests (Pearson and Cochran-Armitage test). We included seasonal and rural variables in the analyses because pesticide usage is predominant in summer and autumn and in rural areas.

An interaction between EDI and exposure was examined but was not significant in multivariable analysis (*p* = 0.93).

Multivariable logistic regression was used to model the relationship between preterm birth prevalence, exposure to atrazine metabolites and neighborhood deprivation taking the available and relevant individual risk factors into account. In order to take the two-level hierarchical structure of the data (mother/neonate pairs and IRIS), and possible intraclass correlation (correlations between individuals belonging to the same geographical entity) into account, we also attempted to apply a mixed model: multilevel model with a random intercept on IRIS [34]. Since there was no specific effect of belonging to a iven IRIS on relative risk of preterm birth (inter-IRIS variance = 0.051, *p* = 0.62), that is to say that there was a lack of variability between the different IRIS units in terms of preterm birth prevalence, we could not apply the mixed model. The results of the logistic random effects model were similar to those of the logistic regression model and are not presented here.

Three nested models were constructed: model 1 with atrazine metabolite exposure adjusted for rural area and season, model 2 (model 1 and individual risk factors: Maternal age, Mother’s occupation, Smoking during pregnancy, Single-parent family, History of preterm birth, Primiparity, Quality of follow-up), model 3 (model 2 and neighborhood deprivation).

We carried out sensitivity analysis separately mother/neonate pairs from 2004 to 2006 and from 2007 to 2010, as LQ changed in 2007, and as usually the global level of atrazine in water distribution systems decrease over time.

The analysis have been done for both degradates (hydroxyatrazine and desethylatrazine) in a separate analysis to see if any effect it might have on the incidence of PTB, but result presentation is restricted to 2-hydroxyatrazine as results were not different.

We carried out the same analysis replacing exposure to atrazine metabolite by exposure to nitrate/atrazine metabolite mixture. Analyses were performed using SAS version 9.3 (SAS Institute Inc., Cary, NC, USA), and the significance level was set at 5% for all analyses.

## 3. Results

**Figure 1:**
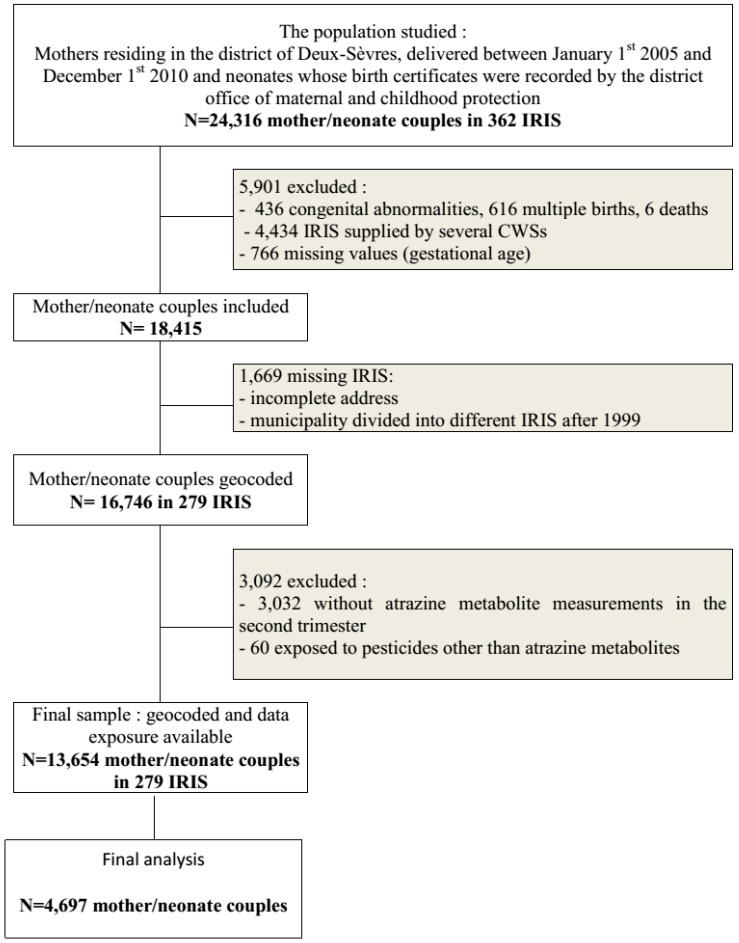
Flow chart of the population for studying the relation between exposure to atrazine metabolite in drinking-water and preterm birth, Deux-Sèvres, France, 2005–2010.

Out of 24,316 pairs we excluded 5091 through exclusion criteria and 69% of the births were successfully geocoded. We also excluded 1669 pairs because of missing IRIS and 3092 because of missing atrazine exposure. Sample included 13,654 mother/neonate pairs (Figure 1), belonging to 279 IRIS and supplied by 51 CWSs (49 ± 49 pairs per IRIS, median 32, from 1 to 307 pairs).

Of the 13,654 pregnant women included in the analyses, 586 (4.3%) had a preterm birth. The rate of preterm birth was 5.2% in non-geocoded pairs and 3.7% in excluded pairs due to missing data on exposure to atrazine metabolites.

Mean birth weight of neonate was 3290 ± 495 g (660–5510 g) and mean gestational age at birth was 39 ± 2 weeks (25–42 weeks). Population characteristics and risk of preterm birth according to population characteristics are presented in Table 1. Pairs excluded for missing IRIS (*n* = 1669) were less exposed than geocoded pairs (*p* < 10^−3^). Among 3092 pairs excluded for missing exposure data, distribution of EDI was different from the final sample: 40% vs. 42% for tertile 1; 42% vs. 38% for tertile 2; 18% vs. 20% for tertile 3 (*p* < 10^−3^).

Neighborhood deprivation and exposure characteristics are presented in Table 1: 39% of the pairs lived in less deprived areas. We have chosen to present results of exposure to 2-hydroxyatrazine during second trimester but results at first trimester are similar (data available from the authors).

In univariable analysis, pairs lived in the most deprived area had higher risk of preterm birth than pairs living in less deprived neighborhoods, but exposure to 2-hydroxyatrazine was not associated with preterm birth (Table 1). History of preterm birth, primiparity and quality of follow up were associated with risk of PTB (Table 1).

Among the 13,654 pairs, the average number of pesticide measurements during second trimester was 1.3 ± 0.5. Average of 2-hydroxyatrazine concentration was 0.019 ± 0.009 (0.014–0.080) µg/L and 0.012 ± 0.009 (0.008–0.100) µg/L for desethylatrazine. Atrazine metabolites concentration decreased over the years, notably in 2007.

Exposure to 2-hydroxyatrazine was more prevalent in the most deprived areas, rural areas and during summer and autumn (Table 2). In multivariable analysis (*n* = 4697), model 2 seemed to be the best model, as EDI did not modify the OR between exposure to 2-hydroxyatrazine and preterm birth (Table 3). Exposure to 2-hydroxyatrazine at a higher concentration than 0.02 µg/L was not associated with an increased risk of preterm birth: ORa 0.945 (95% CI [0.665; 1.343]).

Sensibility analysis with model 2 showed that relationship between exposure to 2-hydroxyatrazine and risk of preterm birth after 2006 was not, but almost, significant: ORa 1.625 95% CI [0.975; 2.710] (Table 4).

Analyses replacing exposure to atrazine metabolite by exposure to nitrate/atrazine metabolite mixture was carried out on 13,539 mother/neonate pairs (since 16,746 pairs in the IRIS involved, 3207 exposure values were missing). Population and EDI characteristics, relationships between population characteristics, EDI, exposure characteristics and preterm birth were the same as in the previous population of 13,654 pairs studied for single exposure to atrazine metabolites. When adjusting on confounders, relationships between exposure characteristics and preterm birth remain non-significant (Table 5).

## 4. Discussion

This is the first epidemiologic study which focuses on the relationship between exposure to endocrine disruptor mixture in drinking-water and risk of preterm birth. Once we had taken confounders into account, and particularly neighborhood deprivation, we found a non-significant relationship between exposure to atrazine metabolites, or mixture, in drinking water during the second trimester of pregnancy between 2005 and 2010 and prevalence of preterm birth neonates.

To date, only Rinsky et al. found on 71,768 births, 1.26-fold higher risk (95% CI [1.19; 1.32]) between atrazine exposure >0.08 µg/L vs. <0.002 µg/L [17]. Our results are in accordance with the majority of studies which found no association. Villanueva et al. found on 3510 births 1.93 (95% CI [0.85; 4.35]) fold higher risk between exposure >0.04 µg/L vs. <0.03 µg/L whereas there were not adjusted for confounding factors [19]. Ochoa-Acuna et al. found on 24,154 births, 1.01 (95% CI [0.93; 1.18]) fold higher risk between exposure in the interval [0.06; 0.5] µg/L vs. <0.06 µg/L whereas exposure to atrazine in drinking-water did not specifically involve the second trimester of pregnancy [18]. In a French cohort of mother/neonates pairs, exposure was assessed by urinary concentration. The presence versus absence of quantifiable levels of atrazine metabolite was associated with fetal growth restriction: ORa 1.5 95% CI [1.0; 2.2] but preterm birth was not an outcome [35].

The definition of our exposure variables may not reflect the precise, actual and global exposure of pregnant mothers, as we explained previously [30]. For the main objective, we preferred to use the multiple imputation method rather to define exposure to atrazine metabolite as a binary variable according to the LQ or use substitution methods which entail bias [31]. This method has resulted in imputing concentrations of mother/neonate pairs with concentrations under LQ, that is to say pairs with concentrations between the LD and the LQ, but also pairs with concentrations under the LD, which are unknown. We were unable to distinguish these categories of pairs because laboratories do not provide data between LD and LQ as it is not necessary to conclude that concentrations are above the regulatory limit (which is 0.10 µg/L for each pesticide) [36]. We may have overestimated exposure for mother/neonate pairs with concentrations under LD which entail underestimation of the relationship between exposure to atrazine metabolites and preterm birth, both in advantaged women and disadvantaged women.

Moreover, as we calculated the mean atrazine metabolite concentration for each trimester among several samples (from 1 to 34), infrequent monitoring could have entailed bias. Authors then suggest applying multiple imputation to fill in water quality values between measurements in CWSs [37].

As our exposure measure was not an individual estimate, we did not know women’s actual water use habits. A recent study we conducted in Deux-Sèvres on pregnant women (EDDS cohort study), showed that 94% of them drank home tap water and only 48+% drank bottled water) [38]. Moreover, as low income women may drink more bottled water than high income women, because they considered that their tap water quality is worse [39]; we have maybe overestimated atrazine metabolite exposure of women from most deprived areas. Despite these disadvantages, the temporal spatial variability and limited sample of an individual estimation method enhances the ecological measurement, which is perhaps less accurate but also had less tendency to diminish the relationship between individual estimation and outcome [40]. Facing cocktail effects and windows of susceptibility, exposure assessment stills the most challenging point of epidemiologic studies on atrazine, particularly if they have an ecologic design [16].

In univariable analysis, we found that living in a more deprived area increases 1.283 (95% CI [1.035; 1.591]) fold the risk of preterm birth; this was comparable with the findings of Zeitlin et al: 1.40 (95% CI [1.14; 1.72]) [13]. To study neighborhood effect, we used the smallest French geographic census unit (IRIS) that researcher could use, and the EDI which reflects subjective as well as objective, material and social deprivation [28]. It is more precise because it has been built with area-level data known to be individual fundamental needs, or perceived necessities of life [41]. However, there was a lack of variability between the different IRIS units in terms of preterm birth prevalence. Were the same type of study to be performed on a geographically larger, regional or inter-regional sample, it would undoubtedly improve exploration of the effect of residential context on risk of preterm birth.

A limitation of the study arises from the database and study population. The database was quite exhaustive because certificate issuance is mandatory in France. Selection bias related to participation of more materially and psychologically invested mothers described in prospective cohorts has been avoided [42]. However, the database was incomplete as 517 (2%) of the births declared in Deux-Sèvres were not recorded. If we consider all these unrecorded births as preterm births, the rate of preterm birth in this study becomes closer to the known rate of preterm births in the department (from 4% to 6%) [43]. We are thus likely to have a selection bias that underestimated the rate of preterm birth in this study as well as the relationship between exposure to atrazine metabolites and preterm birth when preterm neonates are more exposed. However, we found the expected relationship between some individual data, such as history of preterm birth, primiparity, quality of follow up and preterm birth.

In the database, we could not geocode 9% of the pairs included, as did Zeitlin et al. [13], and these mothers were less exposed than geocoded mothers. This factor could have introduced an overestimation of exposure and an underestimation of the relationship between exposure to atrazine metabolites and preterm birth.

Data on mother/neonate pairs were limited to the information available on the health certificates at birth. We lacked data on socio-economic factors such as education level that were not recorded in the database. However, we used maternal occupation, which is a good indicator of socio-economic status [44]. As social factors, we used the single-parent status. We lacked complete data on adverse behaviors but the only type of information that could not be used at all was alcohol consumption, whereas other studies contain no information on smoking during pregnancy [13]. The rate of smoking during pregnancy was comparable with the French national survey rate (20% versus 17%) [45]. We also lacked data on nutritional status. However, these nutritional factors contribute less to preterm birth than do material deprivation factors [10]. The remaining data were reliable; they had undergone a quality control through which the procedures employed were validated.

As preterm birth is particularly susceptible to repeated and accumulated stressors, it would be interesting to understand environmental and socioeconomic life course by analyzing EDCs exposure and neighborhood deprivation through time with longitudinally methods [46]. We would have to use another database indicating the duration of the mother's residence at her address.

## 5. Conclusions

Even if we took neighborhood deprivation into account, we could not show a significant relationship between exposure to atrazine metabolites, or atrazine/nitrate mixtures, in drinking-water during the second trimester of pregnancy and preterm birth. The impact of neighborhood deprivation must be more thoroughly investigated with a geographically larger cohort with the aim of obtaining more variability between areas and with a life course approach.

## Figures and Tables

**Table 1:** Prevalence of preterm birth babies according to population characteristics. Univariable analysis, Deux-Sèvres, France, 2005–2010. *n* = 13,654 mother/neonate pairs and 279 IRIS.

	Pairs		Missing Values		Preterm Birth								
	n	(%)	n	(%)	n	%	ORa		95% CI				*p*
**Individual data**													
**Maternal age**													0.375
<20 years	331	(2%)	26	(0.1%)	16	(4.8%)	1.165	[	0.699	;	1.941	]	
20–35 years	11,198	(82%)			468	(4.2%)	1						
>35 years	2099	(15%)			101	(4.8%)	1.159	[	0.930	;	1.445	]	
**Mother’s occupation** ^a^													0.561
Disadvantaged	4218	(31%)	140	(1%)	179	(4.2%)	1						
Moderately advantaged	7575	(56%)			330	(4.4%)	1.129	[	0.845	;	1.508	]	
Advantaged	1721	(13%)			65	(3.8%)	1.160	[	0.885	;	1.522	]	
**Smoking during pregnancy**			7404	(54%)									0.110
no	5012	(80%)			200	(4.0%)	1						
yes	1238	(20%)			62	(5.0%)	1.269	[	0.947	;	1.699	]	
**Single-parent family**			0	(0%)									0.330
no	12,957	(95%)			551	(4.3%)	1						
yes	697	(5%)			35	(5.0%)	1.190	[	0.839	;	1.690	]	
**History of preterm birth**			4547	(33%)									<10^−3^
no	8675	(95%)			374	(4.3%)	1						
yes	432	(5%)			59	(13.7%)	3.511	[	2.618	;	4.708	]	
**Primiparity**			654	(5%)									<10^−3^
no	7439	(57%)			267	(3.6%)	1						
yes	5561	(43%)			284	(5.1%)	1.446	[	1.219	;	1.715	]	
**Quality of follow up** ^b^			2995	(22%)									<10^−3^
high	10,246	(96%)			48	(3.6%)	1						
low	413	(4%)			369	(11.6%)	3.520	[	2.560	;	4.840	]	
**European deprivation index** ^c^													
Tertile 1 (least deprived)	5756	(42%)	0	(0%)	234	(4.1%)	1						0.05
Tertile 2	5202	(38%)			213	(4.1%)	1.008	[	0.384	;	1.218	]	
Tertile 3 (most deprived)	2696	(20%)			139	(5.2%)	1.283	[	1.035	;	1.591	]	
**Exposure to atrazine metabolites during second trimester**													
**2-hydroxy-atrazine**													
0.013 µg/L	7145	(52%)	0	(0%)	299	(4.2%)	1						0.642
0.013–0.02 µg/L	585	(4%)			29	(5.0%)	1.194	[	0.808	;	1.765	]	
>0.02 µg/L	5924	(43%)			258	(4.4%)	1.043	[	0.879	;	1.236	]	
**Exposure to nitrate during second trimester**													
**Nitrates**													
<16.1 mg/L	4493	(33%)	0	(0%)	186	(4.0%)	1						0.433
16.1–27.2 mg/L	4508	(33%)			209	(5.0%)	1.126	[	0.920	;	1.378	]	
>27.2 mg/L	4480	(33%)			187	(4.0%)	1.009	[	0.820	;	1.241	]	

^a^ more advantageous occupation of either of the parents: Advantaged household: managers or executives, Moderately advantaged household: self-employed, employees and farmers, Disadvantaged household: workers and unemployed; ^b^ at least three ultrasound examinations were done AND seven antenatal care examinations during pregnancy (before 2006) or first antenatal care examination took place during first trimester (after 2006); ^c^ EDI score Tertile 1: <−1.897; Tertile 2: [−1.897; 0.53519]; Tertile 3: >0.53519; 95% CI: confidence interval at 95%.

**Table 2:** Relation between exposure to 2-hydroxyatrazine during second trimester of pregnancy and European deprivation index. Univariable analysis, Deux-Sèvres, France, 2005–2010. *n* = 13,654 mother/neonate pairs and 279 IRIS.

			2-hydroxyatrazine Exposure during Second Trimester						
			0.013 µg/L		0.013–0.020 µg/L		>0.020 µg/L		
	Pairs n (%)		n	%	n	%	n	%	*p*
**European deprivation index**									0.004
Tertile 1 (least deprived)	5756	(42%)	3079	(53.5%)	221	(3.8%)	2456	(42.7%)	
Tertile 2	5202	(38%)	2664	(51.2%)	262	(5.0%)	2276	(43.8%)	
Tertile 3 (most deprived)	2696	(20%)	1402	(52.0%)	102	(3.8%)	1192	(44.2%)	
**Live in rural area**									<10^−3^
no	9505	(70%)	5265	(55.4%)	282	(3.0%)	3958	(41.6%)	
yes	4149	(30%)	1880	(45.3%)	303	(7.3%)	1966	(47.4%)	
**Season during second trimester**									<10^−3^
summer	3377	(25%)	1703	(50.4%)	191	(5.6%)	1483	(43.9%)	
autumn	3696	(27%)	1550	(41.9%)	19	(0.5%)	2127	(57.5%)	
winter	2890	(21%)	1751	(61.6%)	97	(3.4%)	1042	(36.1%)	
spring	3691	(27%)	2141	(58.0%)	278	(7.5%)	1272	(34.5%)	

**Table 3:** Relation between exposure to 2-hydroxyatrazine in drinking-water during second trimester of pregnancy and prevalence of preterm birth babies. Multivariable analysis, Deux-Sèvres, France, 2005–2010.

Police 9	Preterm Birth Risk																				
	Model 1 (*n* = 13,654)							Model 2 (*n* = 4697)							Model 3 (*n* = 4697)						
	ORa		95% CI				*p*	ORa		95% CI				*p*	ORa		95% CI				*p*
**Exposure data during second trimester**																					
**2-hydroxyatrazine**																					
0.013–0.020 µg/L vs. 0.013 µg/L	1.168	[	0.786	;	1.736	]	0.706	0.938	[	0.421	;	2.090	]	0.944	0.929	[	0.666	;	1.345	]	0.944
>0.020 µg/L vs. 0.013 µg/L	1.042	[	0.876	;	1.239	]		0.945	[	0.665	;	1.343	]		0.946	[	0.705	;	1.412	]	
**Live in rural area**: yes vs. no	1.058	[	0.884	;	1.266	]	0.540	1.301	[	0.948	;	1.784	]	0.103	1.316	[	0.949	;	1.827	]	0.100
**Season**: autumn vs. summer	0.854	[	0.678	;	1.074	]	0.462	0.662	[	0.433	;	1.012	]	0.217	0.662	[	0.433	;	1.012	]	0.217
**Season**: winter vs. summer	0.923	[	0.726	;	1.173	]		1.007	[	0.675	;	1.502	]		1.006	[	0.674	;	1.501	]	
**Season**: spring vs. summer	0.852	[	0.678	;	1.071	]		0.891	[	0.611	;	1.301	]		0.883	[	0.605	;	1.290	]	
**Individual data**																					
**Maternal age**																					
<20 vs. 20 to 35 years								1.022	[	0.448	;	2.331	]	0.176	0.983	[	0.430	;	2.247	]	0.1611
>35 vs. 20 to 35 years								1.453	[	0.981	;	2.150	]		1.465	[	0.990	;	2.170	]	
**Mother’s occupation ^a^**																					
Disadvantaged vs. Advantaged								1.280	[	0.697	;	2.351	]	0.726	1.194	[	0.646	;	2.207	]	0.839
Moderately advantaged vs. Advantaged								1.202	[	0.677	;	2.136	]		1.183	[	0.665	;	2.101	]	
**Smoking during pregnancy**: yes vs. no								1.076	[	0.746	;	1.551	]	0.696	1.075	[	0.745	;	1.550	]	0.701
**Single-parent family**: yes vs. no								0.827	[	0.433	;	1.579	]	0.565	0.783	[	0.409	;	1.500	]	0.461
**History of preterm birth**: yes vs. no								5.946	[	3.723	;	9.497	]	<10^−3^	5.890	[	3.686	;	9.414	]	<10^−3^
**Primiparity**: yes vs. no								2.099	[	1.473	;	2.992	]	<10^−3^	2.089	[	1.465	;	2.979	]	<10^−3^
**Quality of follow-up**: low vs. high								4.606	[	2.566	;	8.268	]	<10^−3^	4.487	[	2.494	;	8.074	]	<10^−3^
**European deprivation index (EDI)**																					
Tertile 2 vs. Tertile 1 (least deprived)															0.998	[	0.705	;	1.412	]	0.204
Tertile 3 (most deprived) vs. Tertile 1															1.363	[	0.929	;	2.000	]	

^a^ more advantageous occupation of mother: Advantaged mother: managers or executives, Moderately advantaged mother: self-employed, employees and farmers, Disadvantaged mother: workers and unemployed; 95% CI: confidence interval at 95%.

**Table 4:** Relation between exposure to 2-hydroxyatrazine in drinking-water during second trimester of pregnancy and prevalence of preterm birth babies. Multivariable analysis, Deux-Sèvres, France, 2007–2010 (2005–2006 excluded).

Police 9	Preterm Birth Risk													
	Model 1 (*n* = 8735)							Model 2 (*n* = 3806)						
	ORa		95% CI				*p*	ORa		95% CI				*p*
**Exposure data during second trimester**														
**2-hydroxyatrazine**														
0.013–0.020 µg/L vs. 0.013 µg/L	1.162	[	0.779	;	1.733	]	0.504	0.968	[	0.432	;	2.169	]	0.171
>0.020 µg/L vs. 0.013 µg/L	1.164	[	0.863	;	1.570	]		1.625	[	0.975	;	2.710	]	
**Live in rural area**: yes vs. no	1.076	[	0.859	;	1.347	]	0.526	1.228	[	0.860	;	1.754	]	0.258
**Season**: autumn vs. summer	0.750	[	0.564	;	0.998	]	0.051	0.683	[	0.437	;	1.067	]	0.390
**Season**: winter vs. summer	0.794	[	0.592	;	1.064	]		0.913	[	0.588	;	1.418	]	
**Season**: spring vs. summer	0.693	[	0.524	;	0.916	]		0.824	[	0.545	;	1.245	]	
**Individual data**														
**Maternal age**														
<20 vs. 20 to 35 years								1.167	[	0.502	;	2.713	]	0.255
>35 vs. 20 to 35 years								1.426	[	0.931	;	2.184	]	
**Mother’s occupation** ^a^														
Disadvantaged vs. Advantaged								1.069	[	0.541	;	2.111	]	0.820
Moderately advantaged vs. Advantaged								0.951	[	0.497	;	1.819	]	
**Smoking during pregnancy**: yes vs. no								1.046	[	0.702	;	1.557	]	0.826
**Single-parent family**: yes vs. no								0.746	[	0.378	;	1.473	]	0.399
**History of preterm birth**: yes vs. no								6.185	[	3.677	;	10.405	]	<10^−3^
**Primiparity**: yes vs. no								1.875	[	1.285	;	2.736	]	0.001
**Quality of follow-up**: low vs. high								4.807	[	2.544	;	9.082	]	<10^−3^

^a^ more advantageous occupation of mother: Advantaged mother: managers or executives, Moderately advantaged mother: self-employed, employees and farmers, Disadvantaged mother: workers and unemployed; 95% CI: confidence interval at 95%.

**Table 5:** Relation between exposure to nitrates/atrazine metabolite mixture in drinking-water and prevalence of preterm birth babies. Deux-Sèvres, France, 2005–2010, *n* = 4625 mother/neonate pairs and 277 IRIS.

	Preterm Birth Risk																				
	Model 1 (*n* = 13,481)							Model 2 (*n* = 4625)							Model 3 (*n* = 4625)						
	ORa	95% CI					*p*	ORa	95% CI					*p*	ORa	95% CI				*p*	
Exposure data during second trimester																					
**Exposure to nitrates and atrazine metabolites** ^a^																					
P0N2 vs. P0N1	1.289	[	0.996	;	1.668	]	0.454	0.890	[	0.552	;	1.433	]	0.380	0.861	[	0.529	;	1.404	]	0.383
P0N3 vs. P0N1	1.093	[	0.836	;	1.428	]		0.752	[	0.461	;	1.226	]		0.755	[	0.461	;	1.235	]	
P1N1 vs. P0N1	1.211	[	0.899	;	1.630	]		1.305	[	0.747	;	2.281	]		1.297	[	0.740	;	2.274	]	
P1N2 vs. P0N1	1.079	[	0.657	;	1.771	]		1.103	[	0.559	;	2.179	]		1.077	[	0.542	;	2.138	]	
P1N3 vs. P0N1	1.196	[	0.822	;	1.739	]		1.044	[	0.577	;	1.892	]		1.062	[	0.586	;	1.925	]	
**Live in rural area**: yes vs. no	1.077	[	0.895	;	1.297	]	0.430	1.196	[	0.858	;	1.669	]	0.291	1.198	[	0.842	;	1.703	]	0.315
**Season**: autumn vs. summer	0.841	[	0.669	;	1.058	]	0.399	0.654	[	0.427	;	1.003	]	0.143	0.653	[	0.426	;	1.002	]	0.140
**Season**: winter vs. summer	0.907	[	0.710	;	1.158	]		1.084	[	0.721	;	1.629	]		1.086	[	0.723	;	1.632	]	
**Season**: spring vs. summer	0.842	[	0.668	;	1.061	]		0.935	[	0.634	;	1.377	]		0.931	[	0.631	;	1.373	]	
**Individual data**																					
**Maternal age**																					
<20 vs. 20 to 34 years								1.001	[	0.476	;	2.527	]	0.135	0.967	[	0.422	;	2.216	]	0.123
>34 vs. 20 to 34 years								1.494	[	0.888	;	1.993	]		1.507	[	1.017	;	2.234	]	
**Mother’s occupation** ^b^																					
Disadvantaged vs. Advantaged								1.269	[	0.692	;	2.329	]	0.743	1.187	[	0.643	;	2.191	]	0.834
Moderately advantaged vs. Advantaged								1.206	[	0.680	;	2.138	]		1.185	[	0.668	;	2.101	]	
**Smoking during pregnancy**: yes vs. no								1.069	[	0.739	;	1.545	]	0.723	1.070	[	0.740	;	1.548	]	0.717
**Single-parent family**: yes vs. no								0.809	[	0.424	;	1.545	]	0.521	0.770	[	0.402	;	1.474	]	0.430
**History of preterm birth**: yes vs. no								6.055	[	3.778	;	9.704	]	<10^−3^	6.014	[	3.750	;	9.644	]	<10^−3^
**Primiparity** : yes vs. no								2.157	[	1.509	;	3.084	]	<10^−3^	2.148	[	1.502	;	3.071	]	<10^−3^
**Quality of follow up** ^c^ : high vs. low								4.709	[	2.616	;	8.475	]	<10^−3^	4.590	[	2.543	;	8.283	]	<10^−3^
**European deprivation index**																					
Tertile 2 vs. Tertile 1 (least deprived)															1.006	[	0.703	;	1.438	]	0.232
Tertile 3 vs. Tertile 1 (least deprived)															1.354	[	0.914	;	2.006	]	

^a^ unexposed to atrazine metabolites but exposed to the first tercile of mean nitrate concentrations (P0N1), unexposed to atrazine metabolites but exposed to the second tercile of mean nitrate concentrations(P0N2), unexposed to atrazine metabolites but exposed to the third tercile of mean nitrate concentrations (P0N3), exposed to atrazine metabolites and to the first tercile of mean nitrate concentrations (P1N1), exposed to atrazine metabolites and to the second tercile of mean nitrate concentrations (P1N2), exposed to atrazine metabolites and to the third tercile of mean nitrate concentrations (P1N3); ^b^ more advantageous occupation of either of the parents: Advantaged household: managers or executives, Moderately advantaged household: self-employed, employees and farmers, Disadvantaged household: workers and unemployed; ^c^ at least three ultrasound examinations were done AND seven antenatal care examinations during pregnancy (before 2006) or first antenatal care examination took place during first trimester; 95% CI: confidence interval at 95%.
